# Selenium in the Environment, Metabolism and Involvement in Body Functions 

**DOI:** 10.3390/molecules18033292

**Published:** 2013-03-13

**Authors:** Youcef Mehdi, Jean-Luc Hornick, Louis Istasse, Isabelle Dufrasne

**Affiliations:** 1 ULg-FMV, Nutrition Unit, Department of Animal Production, Boulevard de Colonster 20, Bât. B43 4000, Liège, Belgium; E-Mails: ymehdi@doct.ulg.ac.be (Y.M.); jlhornick@ulg.ac.be (J.-L.H.); listasse@ulg.ac.be (L.I.); 2 ULg-FMV, Station Expérimentale Chemin de la Ferme 6, Bât. B39 4000, Liège, Belgium

**Keywords:** selenium, selenoprotein, environment, antioxidant, seleniummetabolism, deficiency, reproduction

## Abstract

Selenium (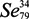) is a metalloid which is close to sulfur (S) in terms of properties. The Se concentration in soil varies with type, texture and organic matter content of the soil and with rainfall. Its assimilation by plants is influenced by the physico-chemical properties of the soil (redox status, pH and microbial activity). The presence of Se in the atmosphere is linked to natural and anthropogenic activities. Selenoproteins, in which selenium is present as selenocysteine, present an important role in many body functions, such as antioxidant defense and the formation of thyroid hormones. Some selenoprotein metabolites play a role in cancer prevention. In the immune system, selenium stimulates antibody formation and activity of helper T cells, cytotoxic T cells and Natural Killer (NK) cells. The mechanisms of intestinal absorption of selenium differ depending on the chemical form of the element. Selenium is mainly absorbed in the duodenum and caecum by active transport through a sodium pump. The recommended daily intake of selenium varies from 60 μg/day for women, to 70 μg/day for men. In growing ruminants the requirements are estimated at 100 μg/kg dry matter and 200 μg/Kg for pregnant or lactating females. A deficiency can cause reproductive disorders in humans and animals.

## 1. General Overview

Selenium is a trace element which is found in small amounts in the organism. It was first isolated in 1817 by the Swedish chemist Jacob Berzelius Jöns and has long been recognised for its toxicity. The importance of selenium was highlighted in 1957. It is a major structural component of many enzymes such as glutathione peroxidase, thioredoxin reductase and deiodinases. These enzymes play important roles in antioxidation, reproduction, muscle function and tumors prevention. It is important that the recommended daily intake of selenium be covered by its intake to ensure proper operation of the functions which it occurs. It is thus worth to knowing its behavior and the various transformations to which it is subject in the body. This article reviews the physicochemical properties of selenium, its presence in the environment, its roles and implications in various body functions. It also reviews the modes of assimilation, excretion and storage of selenium and the possible impact of a deficiency.

### 1.1. Physicochemical Properties of Selenium and Its Compounds

Selenium (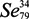) is a metalloid of the same family as oxygen and sulfur (S). The name is derived from Selene—goddess of the moon, by reference to the fact that it is always linked to tellurium, metalloid initially appointed by reference to the Earth [1]. Six isotopes coexist in Nature. Their mass numbers are very close to 74, 76, 77, 78, 80 and 82 [2]. It resembles S in terms of atomic size, bond energies, ionization potentials and main oxidation states [3].

Selenium is a semi metal and it consequently possesses intermediate properties between a metal and a non-metal. It is stable and does not oxidize at ordinary temperatures. When it burns, it produces a blue flame and selenium dioxide. This reaction is accompanied by a characteristic and unpleasant odour. Selenium can be combined with many elements (hydrogen, fluorine, chlorine, bromine, phosphorus, *etc*.). It thus forms compounds with a close analogy to those of sulfur [4,5,6].

The affinity of selenium for oxygen is lower than that of sulfur. Only two oxides, SeO_2_ and SeO_3_, are well known. Dioxide is formed by the combustion of selenium in air. This is a stable product that dissolves in water, giving selenious acid (H_2_SeO_3_). The solution obtained can oxide the majority of metals, except gold, platinum and palladium.

Selenic acid (H_2_SeO_4_) is a strong and hygroscopic diacid. It is more oxidizing than H_2_SO_4_. It is obtained by the action of a powerful oxidizing agent (fluorine, chlorine, bromine, permanganate ion, anodic oxidation...) with Se, SeO_2_, or H_2_SeO_3_ and the presence of water.

Hydrogen selenide (H_2_Se) is released during the reaction of hydrogen with selenium (400 °C), or the reaction of water (or acids) with the metal selenides. H_2_Se is a highly reactive compound. It starts to decompose in Se and H_2_ at 160 °C. It also decomposes quickly in moist air and forms a deposit of red selenium [6,7]. 

### 1.2. The Physical and Chemical Forms of Selenium

**Figure 1:**
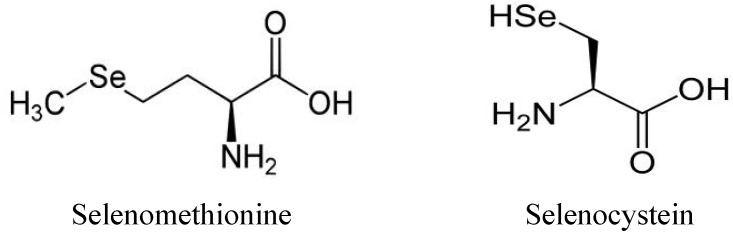
Selenomethionine and selenocysteine the main of organic forms of selenium.

Selenium is present in Nature and in organisms as organic and/or inorganic forms. The main organic forms are selenomethionine (Semet) and selenocysteine (Secys). Figure 1 illustrates the organic forms of selenium. The inorganic forms are selenite (SeO_3_^−2^), selenide (Se^2^^−^), selenate (SeO_4_^−2^) and the selenium element (Se) [8].

At ordinary temperature selenium is a solid substance [6]. Like sulfur, selenium takes various physical forms [5,9]. The amorphous selenium—a red-brick powder—is obtained by precipitation from aqueous solution. For example, it is obtained by reduction of a solution of selenious acid by hydrogen, zinc or sulfur dioxide. Its density is 4.26. It is endowed with photoconductive properties. It turns gray at an ill-defined temperature between 110 and 180 °C. Vitreous selenium is a brown and presenting as vitreous amorphous mass. It is formed by rapid cooling of liquid selenium. Selenium gray is a variety thermodynamically stable. It may be obtained by slow cooling of liquid selenium. Its density is 4.80. It is used for its semiconducting properties.

### 1.3. Use and Production

Selenium is found in Nature in pyrites of copper and iron, sulphide ores of copper, lead, nickel, gold or silver. It is encountered in these compounds at variable levels, between 0.1 and 2 ppm. It is also found in oil, where it can reach a concentration of 0.8 ppm. It is used by humans in a variety of industrial and medical applications. Global production of selenium is estimated to be between 2,500 and 2,800 tons per year. Japan (551 tons), Canada (384 t), Belgium (200 t) and Germany (100 t) are the main producers. The United Kingdom, Finland, Belgium and Germany are the major producers and importers of selenium in Europe [6,7]. It is a byproduct of metallurgy. It is obtained from sludge electrolytic refining of copper. The sludge contains a proportion of 5 to 25% of selenium. Roasting the sludge with soda crystals, or in a sulfuric acid medium allows its extraction. This is the most profitable production for industries. It is also produced from the reprocessing of residues from the electrolysis of lead and nickel [2,6,7]. 

Compounds commonly used in industry are selenium dioxide, selenite and sodium selenate. Industrial applications for this metalloid and its compounds can be divided into various categories. Thirty percent of applications are for the electrical and electronic fields. Selenium is used in the industry of rectifier currents, photo cells, drums photocopiers, *etc*. Some selenium compounds are used as pigments and additives for lubricating oils in the paint industry (19%). They are also used in the glass industry and ceramics (20%) for discoloration and pigmentation. In metallurgy an amount of 14% of the total selenium is used to prepare easily machinable alloys, and provide resistance to corrosion and for the surface treatment of metals. Eleven percent of applications are registered in various other industries. It is used for the vulcanization of rubber in the chemical industry and for the oxidation of catalysts. It is also involved in the manufacture of pharmaceutical products for human and veterinary purposes, as s dietary supplement and in the treatment of dandruff, seborrheic dermatitis and other skin diseases. Selenium is also used in the fields of agriculture and biology (6%), to amend deficient soil, in insecticides and ultimately, in animal feeding [6,10].

## 2. Sources of Selenium in the Environment and its Location

Most plant and animal tissues contain traces of selenium [11]. It is widespread in the Earth’s crust where average concentration is 0.09 mg·kg^−1^. 

### 2.1. In Soils

In soils, selenium occurrence is mainly due to the erosion of rocks containing selenites and selenides which are associated with sulphide minerals and with mass fractions less than 1 mg/kg. Selenium is found in soils in the form of elemental selenium, such as selenate salts and ferric selenite or in its organic form. Selenite (SeO_3_^2−^) and selenate forms (SeO_4_^2^^−^) are common in most soils. These anionic forms are highly soluble, mobile, bio-available and potentially toxic. Organic forms come mainly from the decomposition of plants that accumulate selenium [12,13].

The selenium in soil varies with soil type and texture, organic matter content and with rainfall. Its assimilation by the plant is influenced by the physicochemical factors of the soil, such as redox status, pH and microbiological activity. The average concentration of selenium in soil varies from 0.1 to 0.7 mg·kg^−1^. For clay soils, it is 0.8 to 2 mg·kg^−1^, while in tropical soils, it is 2 to 4.5 mg·kg^−1^ [7]. Volcanic soils and granite are poor in selenium. These soils are found in the mountainous countries of Northern Europe, such as Finland, Sweden and Scotland. Shale soils are rich in selenium. Generally, selenium tends to be concentrated in soils of the driest regions in the world. The toxic effects of selenium on animals occur on these soils [14,15]. Soil acidity determines the rate of selenium in plants and crops. Alkaline soils release more selenium than acid ones. In alkaline soils, selenite oxidizes and becomes soluble selenate, which is easily assimilated by the plant. By contrast, in acid soils, selenite is often linked to iron hydroxides, which makes it highly fixed by the soil [16].

### 2.2. Plant Sources

Selenium concentrations in plants are related to selenium levels in the surrounding soils. Incorporation and redistribution of selenium by the roots occurs rapidly, but is dependent on the species and physiological conditions of the plant. In most cases, 85% of selenate and 70% of selenite are found in the aerial tissues [17]. The normal content of selenium in forages ranges from 0.1 to 0.5 ppm. The risk of livestock poisoning becomes high beyond 5 ppm [18]. There are seleniferous plants, selenium accumulating plants and others plants with an average content of selenium. Seleniferous plants are characterized by a high content of selenium. This is observed for some plants growing in arid regions of China and the United States, where selenium accumulates up to 20,000 ppm [14]. There are over twenty such accumulating plants. Some species such as *Astragalus* (*A. bisulcatus*, *A. racemosus*, *A. pectinatus*, *A.* *thephorosides*, *A. praelongus*) can accumulate several thousand ppm of selenium. *Machaeranthera* and *Oonoposis* contain 800 ppm. *Stanleya* and *Haplopappas* can contain 700 and 120 ppm, respectively. Plants with an average content of selenium are toxic to animals. This is the case for the *Aster*, the *Gutierrezia* and *Atriplex*, which contain 72, 60 and 50 ppm respectively [19]. According to Minson [20], grasses contain typically higher concentrations of selenium than leguminous plants. This difference decreases in soils with low levels of selenium. Cereal plants can also store selenium in the seeds, mainly in the form of selenomethionine. Levels vary greatly, depending on the region, from 0.006 ppm in DM in the deficient areas of Sweden and New Zealand, to 3.06 ppm in some parts of Canada [15,21]. Table 1 shows the concentration of selenium in some plants and animals foods. 

**Table 1:** Seleniumcontentof various animal foods.

Foods	Average content (mg/kg DM)
Trial conducted in France [22]	
Meadow grass	0.24
Alfalfa	0.23
Peas fodder	0.32
Maize silage	0.16
Fresh grass silage	0.19
Meadow hay	0.14
Alfalfa hay	0.37
Straw	0.16
Barley	0.09
Oat	0.14
Wheat	0.11
Soya bean meal	0.40
Peanut seed meal	0.32
Rapeseed meal	0.15
Urea	0.10
Dried sugar beet pulp	0.16
Green wheat	0.30
Trial conducted in Southern Belgium [23]	
Lolium perenne Elgon	0.055
Lolium perenne Ritz	0.269
Trifolium pratense	0.090
Rumex acetosa	0.463
Plantago major	0.631
Plantago lanceolata	0.288
Sanguisorba officinalis	0.605
Knautia arvensis	0.123
Trial conducted in Switzerland [24]	
Grass	0.026
Grass silage	0.054
Hay	0.034
Silage but	0.018
fodder beet	0.026
Compound feedstuffs for dairy cows	0.02–0.79

Selenate is taken up by plants ten times more than selenite [17]. These two compounds are metabolized in chloroplasts by the same metabolic pathways as sulfur because of the chemical similarities between the two elements. Selenate is first activated by ATP sulfurylase-adenosine 5'-phosphosélénate (APSE), then it is reduced by adenosine 5'-phosphosulfate reductase into selenite, and the latter is non-enzymatically reduced to selenide by glutathione. 

**Figure 2:**
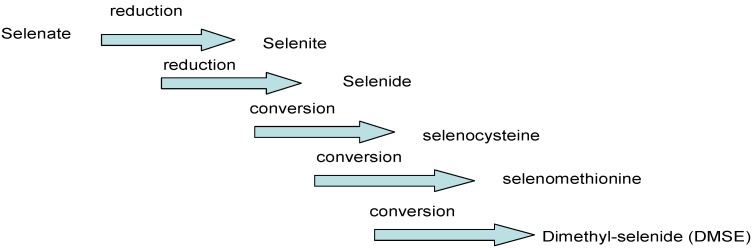
Formation of dimethyl-selenide [(CH3)_2_Se] in not accumulating selenium plants [25].

There are two types of potential metabolic processes, depending on the accumulative capacity of the plant. For non-accumulating plants, mechanisms leading to the formation of dimethylselenide (DMSE) can be described by five major steps as illustrated in Figure 2. The mechanism differs in plants accumulating selenium, after the formation of selenocysteine, which would be bi-methylated to form dimethyl diselenide (DMDSe) [25,26]. 

### 2.3. Selenium in Water

Selenium is also found in water. It originates from atmospheric deposits or soil drainage and sub-soils which are naturally rich in selenium. The concentration in water varies from a few to several hundred mg.L^−^^1^. In most cases it does not exceed 10 mg·L^−1^. Its concentration in sea water varies from 0.04 to 0.12 g·L^−1^. Selenium concentration in groundwater is estimated at 0.12 μg.L^−^^1^ in Brussels (Belgium). It varies from 2.4 to 40.5 μg.L^−^^1^ in France according to the areas. In drinking water, the concentration is 10 μg·L^−1^. Such concentration is the lower limit recommended by the World Health Organization [13,27]. In most cases, high concentrations are due to supplementation of agricultural land with fertilizers containing selenium.

In surface waters, selenide and selenate sodium predominate. In freshwater selenium is present mainly as selenate and selenite. Selenite is adsorbed easily on suspended solids. Selenides and selenates are highly soluble and very mobile. Selenium may also be present as methylated and volatile organic species whose production is favored by microorganisms and microalgae. In water organisms, it may be linked to different proteins and enzymes [7,28].

### 2.4. Sources of Selenium in the Air

The atmosphere plays an important role in the biogeochemical cycling of selenium. It influences the transport and transformation. The presence of selenium is linked to natural activities such as soil erosion, volcanism and forest fires. It is also related to human activities like burning fossil fuels and incineration of garbage, tires and paper. Burning coal and oil are the primary sources of emissions of selenium compounds in the air. The selenium content in ambient air is generally low. It varies from 1 to 10 ng·m^−3^ [13,29]. Three groups of selenium compounds can be distinguished in the atmosphere according to their behaviour: volatile organic compounds (DMSe, DMDSe and methaneselenol), volatile inorganic compounds (selenium dioxide), and elemental selenium, linked to ashes or particles. Dimethyl selenide is a stable compound. Hydrogen selenide and selenium dioxide are unstable in air. Hydrogen selenide is oxidized into selenium and H_2_O. Selenium dioxide is transformed into selenious acid in moist conditions [30].

### 2.5. Food and Feed Sources of Selenium

The selenium content of grains and vegetables generally depends on the selenium content in the corresponding soils. Vegetables such as turnips, peas, beans, carrots, tomatoes, beets, potatoes and cucumbers contain a maximum of 6 mg·g^−1^ of selenium, even when they are grown on seleniferous soil. Vegetables such as onions and asparagus may accumulate up to 17 μg·g^−1^ of selenium when they are grown in such soils. Garlic and brassicas (cabbage, broccoli, mustard ...) are also able to effectively accumulate selenium. Fruits generally contain only low amounts of selenium, rarely exceeding 10 µg·kg^−1^. Brazil nuts have high levels of protein and are known for their very high concentrations of selenium [31,32,33]. Similarly, the selenium content of foods from animal sources varies according the diet of these animals. Table 2 gives the contents of selenium in some human feeds. The major selenium form is selenomethionine. It is associated with negligible amounts of selenocysteine and selenite. The usual forms of oral supplementation are sodium selenite, sodium selenate, potassium selenate and barium selenate [8].

**Table 2:** Selenium contentof selenium insomehuman feeds.

Feeds	Se content
Tinggi [3]	(mg·kg^−1^ FM)
Cereal, cereal products	0.01–0.31
Bread	0.06–0.15
Rice (white)	0.05–0.08
Pasta/spaghetti	0.01–0.10
Meat and meat products	0.06–0.34
Chicken	0.081–0.142
Pork	0.032–0.198
Beef	0.042–0.142
Lamb	0.033–0.260
Milk and dairy products	<0.001–0.11
Fairweather-Tait *et al*. [34]	(mg·kg^−1^ DM)
Onions	<0.5
Lentils	0.24–0.36
Potatoes	0.12
Crustaceans	0.36–1.33
Cod	1.5
Tuna	5.6

## 3. Role of Selenium in the Body

Selenium is an essential component of selenoproteins playing an important role in many biological functions, such as antioxidant defense, formation of thyroid hormones, DNA synthesis, fertility and reproduction. Selenium can be converted in the organism into various metabolites. Some, like methylselenol, play a role in cancer prevention. Selenium has also a role, besides vitamin E, in muscle function by improving endurance and recovery and slowing the ageing process [35,36].

Thirty selenoproteins have been identified in recent years throughout 25 mammalian genes. Selenocysteine is present in selenoprotein once per subunit except Selenoprotein-P (SelP) wich contain 10 (humain, rat) or 12 (bovine) secys in its polypeptides chain [37,38]. 

### 3.1. Selenoproteins

#### 3.1.1. Glutathione Peroxidase (GPx)

The glutathione peroxidases (GPx) are a family of antioxidant enzymes. Their main function is to neutralize the hydrogen peroxide and organic hydroperoxides in the intracellular and extracellular compartments. In a recent review, Brigelius *et al*. [39] summarized the latest knowledge on various aspects of glutathione peroxidases. There are eight forms of GPx which are characterized by similar features. They have different modes and sites of action and different chemical forms. They protect cells, in synergy with vitamin E, from the accumulation of H_2_O_2_ or organic hydroperoxydes and they ensure the continued integrity of cell membranes. Their enzymatic activity is directly proportional to selenium intake, especially for forms 1 to 4 which are dependent on selenium, in order to perform neutralization. There is, therefore, a strong link between selenium deficiency and oxidative stress [40,41,42].

Glutathione peroxidase-1 (GPx-1) is widespread throughout the whole body. It is expressed at very high levels in erythrocytes, liver, kidneys and lungs [43]. Its main activity is antioxidant. It is the first enzyme to be affected in the case of selenium deficiency [34,44]. Glutathione peroxidase-2 (GPx-2) is localized predominantly in the gastrointestinal tissues and in the human liver. It protects against oxidative damages and presents 65% analogy with the GPx1 [45]. Glutathione peroxidase-3 (GPx-3) is localized in extracellular fluid and plasma. It represents 10 to 30% of selenium found in plasma. It is found in the liver, kidneys, heart, lungs, thyroid, gastrointestinal tract and breasts, and also in the placenta and the male reproductive system [46]. Its role is antioxidant in the plasma and it can also reduce lipid hydroperoxides. Glutathione peroxidase-4 (GPx-4) is widely spread in the human body. Strong activity is observed in the testes. It is located in cells in the cytosol, mitochondria and nucleus [47]. Besides its antioxidant activity, it protects membranes from peroxidative degradation (an important role is suggested in the brain) [48]. It can convert cholesterol and cholesterol ester hydroperoxides into less toxic derivatives. It protects against DNA damages by oxidation. It plays a role in regulating the 15-lipoxygenase and 5-lipoxygenase pathways. GPx-4 is important for male fertility and maturation, function and sperm motility. Glutathione peroxidase-5 (GPx-5) is present in the embryo and the olfactory epithelium, its role remains unknown [34]. The GPx 6, 7 and 8 are less known. The GPx-6 is a selenoprotein found only in humans, it is a homologue of GPx-3 and its role remains unknown. There is an inverse relationship between GPx-7 and the proliferation of cancer cells. The GPx-7 is located in the lumen of the endoplasmic reticulum. It has an antioxidant function and it is probably involved in protein folding as well as the GPx-8 which is a membrane protein of the endoplasmic reticulum and the last of the family of glutathione peroxidases to be discovered [39]. 

#### 3.1.2. Deiodinases

These three selenoproteins (5'DI, 5'DII, 5'DIII) were the second type of selenoproteins to be characterized. Deiodinase I is found primarily in the liver, kidneys, thyroid and brown fat. It plays a role in thyroid hormone metabolism. It converts inactive thyroxine into active 3,3'-5'triiodothyronine. The deiodinase type II is abundant in the central nervous system, in the brown adipose and in the skeletal muscles. The deiodinase type II also has a role in the activation of thyroid hormones. The deiodinase III has an activity in fetal and in the deactivation of thyroid hormones. It is present in the placenta, uterus, fetus and central nervous system [34,41].

#### 3.1.3. Selenoprotein-P (SelP)

SelP is an extracellular glycoprotein. It was discovered in humans in 1993 [49] and is the most abundant selenoprotein found in plasma. It constitutes more than 50% of plasma selenium reserves [37]. It is highly expressed in the brain, liver and testes. It plays a role in homeostasis and the transport of selenium in tissues [34], and it is also an extracellular antioxidant. It eliminates peroxynitrite, which results from the reaction of superoxide ions with nitric oxide. These two products are radicals produced at sites of inflammation [41].

#### 3.1.4. Thioredoxin Reductase

There are three thioredoxin reductases (TR1, TR2 and TR3). They play an antioxidant role and control the intracellular redox potential. They decrease the concentration of thioredoxin (TR1 and TR2). They also act as cell growth factor in DNA synthesis and inhibition of apoptosis (programmed cell death). The TR1 is located in the intracellular content (cytosolic/nuclear). The TR2 is widespread, especially in the mitochondria. The TR3 is specifically localized in the testes [34,41].

#### 3.1.5. Other Selenoproteins

Table 3 shows the different selenoproteins in humans and their functions.

**Table 3:** Some human selenoproteins and their functions.

Groupe/nom	Abbreviation	Location	Main Functions
Selenoprotein-W	SelW	Prostate, brain, colon, heart and skeletal muscle	Antioxidant in human lung cancer cells, protect the developing myoblast Calcium-binding [34,50]
Selenoprotein-N	SelN	Most tissues, transmembrane glycoprotein associated with endoplasmic reticulum	Proper muscle development. Cell proliferation, redox signalling, calcium homeostasis [51]
Selenoprotein-S	SelS	Plasma membranes, endoplasmic reticulum	Elimination of misfolded proteins from the ER reticulum, regulation of inflammation [52]
Selenoprotein-K	SelK	Spleen, immune cells and endoplasmic reticulum	Possible antioxidant and development activity [53]
Selenoprotein-H	SelH	Spleen, brain, nucleus	Gene regulation of the glutathione synthesis, transcription factor, increasing of cell viability [51,54]
Selenoprotein-R	SelR	Liver, kidney	Antioxidant, methionine metabolism and proteins repair. Reduction of sulfoxymethyl group [51]
Selenoprotein-M	SelM	Endoplasmic reticulum, neuronal cells	Protein folding, antioxidant activity [48,51]
15kDselenoprotein	Sel15	Endoplasmic reticulum	Plays a role in protein folding Protects against cancer? [34,35]
Mitochondrial capsular selenoprotein	MCSeP	Sperm mitochondrial capsule	GPX4 storage [34,35]
Selenophosphate synthetase-2	SPS-2	Kidney, liver, testis	Synthesis of selenophosphate for selenoprotein synthesis, Secys biosynthesis [55,56]

### 3.2. Roles of Selenium in the Immune Response

Selenium can be found in large amounts in the spleen, liver and lymph nodes. Selenium has been showed to stimulate the antibody formation and the activity of the helper T cells along with the cytotoxic T and NK cells. It is also implicated in the stimulation of the phagocytic cells migration and in the phagocytosis [5,57]. In terms of selenium status, some metabolites of selenium and selenoproteins such as GPX1 and TR1 were shown to be involved in the immune and inflammatory responses, the mechanisms responsible for the beneficial effects being not yet fully understood [58,59]. The production of prostaglandins PGI_2_, PGE_2_ and PGF_2α_ was lower in endothelial cells deficient in selenium. Furthermore, in selenium deficient dairy cows, Sordillo [58] reported a decrease in the ability of blood and milk neutrophils to kill pathogens. An opposite situation has been reported in neutrophils from cows with high selenium status.

A link was established between nutritional selenium provision and mastitis frequency in cows, keeping in mind that the phagocytic activity of neutrophils was the primary defense mechanism against mastitis [42]. According Hafnawy [60], selenium supplemented cows were characterized by a high IgG concentration in serum and colostrum. Higher IgG levels in the serum were also recorded in their calves. Neutrophils from these cows showed an improved phagocytic and bactericidal activity against *Candida albicans* and *Staphylococcus aureus*. Similarly, it was reported that *in vitro* selenium supplementation of breast macrophages enhanced the production of neutrophil chemotactic factors upon stimulation with *Staphylococcus aureus* [58].

### 3.3. Cancer and Cardiovascular Diseases

A study by Davis *et al*. [55] showed the involvement of different selenoproteins in the prevention against cancer. Meta-analytic studies of the epidemiological literature showed that selenium deficiency was a cancer promoting factor. Similarly, negative correlations were found between the levels of selenium in the diet or forages and cancer mortality. The authors reported that the risk of cancer was 2–6 times lower in high selenium serum levels compared to low levels (<100 ng/mL), or low selenium intake (<55 μg/day). Davis *et al*. [55] reported that selenium had a protective effect against lung cancer in populations with low selenium status. By contrast in a healthy population, Cortés Jofré *et al*. [61] reported that there was no evidence for the recommendation of selenium alone or in combination with vitamins such as vitamins A, C or E for lung cancer prevention and mortality due to lung cancer. Selenium supplementation in animal models above food requirements was preventive against liver, pancreas, prostate, esophagus and colon cancers. Similarly also, an enriched selenite salt supplementation in a community of 21,000 persons in China reduced liver cancer by 35% [55]. A 200 μg of selenium per day intake during 7 years decreased prostate cancer among participants in a Nutritional Prevention of Cancer (NPC) trial [62]. However, it was noted that the results of the NPC test also showed an increased risk of type 2 diabetes mellitus among participants with plasma selenium concentration in the upper tertile at the beginning of the study. Similarly there were some evidences of selenium anticancer properties derived from studies with rodents in which the β-lyase, an enzyme required for the conversion of selenomethionine to methylselenol, was 800 times higher than in humans. The discrepancies in terms of response between rodents and humans can therefore create differences between clinical and preclinical studies [55].

Selenium concentrations were significantly lower in patients suffering from acute myocardial infractions, selenium deficiency being an etiological factor of the heart failure syndromes (Keshan disease). There was an inverse association between selenium concentrations and coronary heart disease incidences, especially in populations in which the selenium intake or the selenium status was low [56,63]. However, according to Fairweather-Tait *et al*. [56] the observation that low selenium concentrations were associated with cardiovascular risk should be treated as suggestive. Similarly recent reviews [64,65], showed an U-shape response curve between the selenium status and the risk of cardiovascular disease.

In randomized trials, Rayman *et al*. [63] reported that selenium supplementation did not have a protective effect against cardiovascular disease and mortality. By contrast, in a study of the influence of a diet enriched with organic selenium in patients suffering from cardiovascular disease, Derbeneva *et al*. [66] reported positive changes in patients, the changes being associated with an increased activity, improved overall health and improved cognitive functions.

### 3.4. Role of Selenium in Reproduction

Many studies have highlighted the involvement of selenium in human and animal reproduction. Selenium plays an important role in fertility, embryonic implantation, placenta retention, synthesis of testosterone and sperm, and sperm mobility. Selenium deficiency affects reproductive parameters and animal performance. Indeed, many cases of infertility were recorded in selenodeficient areas related to the lack of selenium. Selenium increases fertility in dairy cows [67]. In pastures very poor in selenium, Meschy [42] reported a remarkable increase in fertility (92% *vs.* 45%) with selenium supplementation. Such a result was not found in cases where a supplement of vitamin E, or of another antioxidant, was given. The increase in fertility was attributed to a decrease in embryonic mortality during the first month of pregnancy. 

Selenium plays a specific role during implantation. Selenium supplementation of pregnant ewes improves the viability of lambs with an increase of survival from 0.61 to 0.91 during the first five days. Selenium deprivation also affects viability and hatching in quail. Generally, hatching rate is the parameter most affected in cases of inadequate selenium intake in poultry [35]. In the study of Harrison [68], ovarian cysts were less frequent (19% *vs.* 50%) after an injection of selenium, in dairy cows with deficient diets. The result was not significant with additional vitamin E alone. Selenium deficiencies have also been involved in retained placenta and metritis. Spears [69], reported that selenium supplementation of dairy cows decreased the incidence of retained placenta. Cases of uterine prolapse were attributed to a deficiency of selenium [70]. Moreover, low concentrations of selenium in red blood cells and hairs are recorded in women with recurrent spontaneous abortions [71].

The deficiency is likely to affect male fertility, particularly in the synthesis of testosterone and sperm [72]. According to Maiorino [73], selenium deficiency is most often characterized by fragility of the intermediate piece with as result reduced sperm motility. In 64 men, Mistry [71] reported improvement in semen quality and fertility after selenium supplementation. The study was conducted in Scotland, with placebo control and randomized (RCT). These beneficial effects of selenium supplementation were reported in other RCTs conducted in Tunisia and Iran. This improvement includes the count, concentration, morphology and motility of sperm.

## 4. Metabolism of Selenium

### 4.1. Transformation, Absorption and Transport

Glutathione (GSH) is the main component of the metabolism of selenium. It takes part in a series of reduction reactions. In the case of selenite, these reactions convert it into hydrogen selenide (H_2_Se). The H_2_Se ensures the supply of active selenium for the synthesis of selenoproteins. The H_2_Se undergoes a serie of sequential methylations to give the late trimethylselenonium ion [(CH_3_)_3_Se^+^] [71]. 

The efficiency of intestinal absorption of selenium is much lower in ruminants than in monogastric species. For selenite, the absorption is 79 and 80% in poultry and pork, while it is only 29% in sheep. For selenomethionine and selenate the absorption is greater than 90% in monogastrics and poultry. These differences appear to result from the reduction of selenite and selenate in selenides which are less available in ruminants [42]. 

The preintestinal absorption of selenium is negligible. So, the absorption operates mainly in the duodenum and caecum. Absorption occurs primarily by active transport through a sodium pump. The mechanisms of intestinal absorption of selenium are not well known and appear different depending of the chemical form of the element. Selenite is absorbed by simple diffusion, whereas selenate would be by a cotransport sodium selenate and exchange selenate/OH^−^. Organic forms (selenomethionine, selenocysteine) follow the mechanisms of amino acid uptake. The ingested selenomethionine is absorbed in the small intestine by an active mechanism similar to that used for methionine, which is via the transport system of neutral amino acids Na^+^ [74,75].

Some elements decrease the rate of absorption of selenium. This is the case of sulfur, lead, arsenic, calcium and Fe^+3^. Fe^+3^ precipitates selenium to a complex form unassimilable by the enterocytes. Sulfur decreases the absorption of selenium by steric competitiveness [69,74] at a concentration over 2.4 g·kg^−1^ DM. Similarly, the concentration of hepatic selenium reduces when the sulfur content of the diet is as high as 2.15 to 4.0 g·kg^−1^ DM [76]. The hepatic selenium concentration reflects the level of intestinal absorption. Serum levels of selenium and its content in all tissues decreased in the case of high concentration of lead in the diet of the calf. This decrease is organ-depend [77]. Garcia-Vaquero [78], showed that calcium supplementation in cattle, with concentrations typically used in intensive production, causes a significant decrease in the selenium content in muscle. According to Harrison [68], a calcium level of 0.8% DM in the feed allows an optimal apparent absorption of selenium in dairy cows in late pregnancy.

Selenite is rapidly and selectively taken up by erythrocytes. It is reduced by glutathione and glutathione reductase and transported in plasma in the form of selenide which binds selectively to albumin. It is then transported to the liver [79]. As reported above, selenium is transported by blood in the form of selenoprotein P [42]. Selenium also binds to α and β globulins that have a great affinity for selenium, and to LDL (low density lipoprotein) and VLDL (very low density lipoprotein). One to 2% of selenium in plasma is bound to GSH-Px [80].

### 4.2. Excretion

Seboussi reported that the removal percentage of selenium in the urine depends on the amount of selenium ingested, the chemical form, the composition of the food, the selenium status of the animal and the percentage of the glomerular filtration [81]. Urine is the dominant route of excretion of selenium in monogastrics. In ruminants, the urinary excretion of selenium is generally low. Selenium is predominantly excreted through the feces due to a low intestinal absorption [42]. The selenium content of milk is relatively low (about 0.05 ppm). It increases significantly in the event of dietary supplementation at an average concentration of 0.16 ppm.

### 4.3. Homeostasis

Selenium is set aside in the form of selenomethionine and stored in the organs and tissues with variable density: 30% in liver, 30% in muscle, 15% in kidney, 10% in plasma, and 15% in other organs [71]. The selenium homeostasis is primarily achieved by the reserves of selenomethionine in the kidney and liver. The stored selenium is used when selenium food intake is too low for selenoproteins synthesis [82].

## 5. Nutritional Requirements and Effects of Deficiencies or Excesses in Selenium

### 5.1. In Animals

The requirements for selenium in animals are expressed in terms of dry matter intake density. In France, the National Institute of Agronomic Research (INRA) adopted the concentration value of 100 μg·kg^−1^ DM for ruminants. In Germany, the recommendation are 100 μg·kg^−1^ DM for growing animals and 200 μg kg^−1^ for pregnant or lactating females [42]. Some diseases and disorders related to selenium deficiency are well known in animals and humans. In animals, selenium deficiency is fairly common without supplementary feeding, especially with forages that are grown on neutral or acidic soils. Manifestations of selenium deficiency differ in the young and the adult animals. The first organs affected by selenium deficiency are the heart, the skeletal muscle and the liver.

In young animals, white muscle disease is the most prevalent disorder resulting from selenium deficiency. It is rare and discreet in adults. It is a degenerative myopathy which the predilection sites are the skeletal muscle, the heart and the bird’s gizzard. Striated muscles and hearts undergo a waxy degeneration which deprives them of any features and provides a whitish color. In small ruminants it is called Stiff lamb disease. Most cases occur in the weeks following birth (four months for cattle and two months for small ruminants).

White muscle disease affects poultry, cattle, goats, horses, sheep, pigs and deers. It especially affects animals with high growth rate. Kids are more susceptible than lambs or calves. The main clinical symptoms are musculoskeletal disorders, a position of urination and a tail slightly raised. Muscle tremors, difficulty swallowing and a rapid heart rate can also be observed. Sometimes the disease resulted in a sudden cardiac arrest. Table 4 shows other diseases related to selenium deficiency. In sheep, wool is the most sensitive to selenium production deficiency. In dairy cows, a decrease in the fat content of milk is observed.

Without prevention or treatment of selenium deficiency, reduced performance and even mortality may occur mortality and production cuts may be high. Many methods can be used to prevent deficiencies, such as the use of enriched selenium mineral salts, application a fertilizer with selenium, incorporation of selenium in drinking water, injections, implants and selenium bolus [24,35,42].

The major signs of selenium toxicity are musculoskeletal disorders such as stiff gait and lameness. They are due to alteration of the cartilages. Fast-growing, soft and brittle hooves and hair loss can also be seen in case of excess. These symptoms are quite similar to zinc deficiency making diagnosis difficult [42].

**Table 4:** Summary of specific clinical disorders that respond to selenium supplementation (adapted fromSuttle [35]).

Disorder	Description and consequences	Predilection site	Species affected
Exudative diathesis	Increased capillary permeability: oedema, swelling and bruising	Thorax, neck, wings	Poultry mainly, pig
Hepatosis	Necrosis	Liver	Pig
Mulberry heart disease	Microangiopathy	Heart mainly, brain	Pig

### 5.2. In Humans

The selenium recommended daily intake of the CSS (Council of Health) in Belgium [83] ranges from 60 μg·day^−^^1^ for women to 70 μg·day^−^^1^ for men (from 14 years). This recommendation is increased in to 65 µg for pregnant women and 75 μg during lactation. The European Food Safety Authority (EFSA) 2006 guidelines sets the tolerable upper intake (UL: Tolerable Upper Intake Level) to 300 μg·day^−^^1^ for adults. For children, the tolerable upper intake is 60 µg·day^−^^1^ (children aged 1 to 3 years) to 250 µg·day^−^^1^ (children aged 15 to 17 years).

In humans, papers on symptoms of selenium deficiency have described only extreme, severe and prolonged cases of deprivation. It is characterized by a necrotizing cardiomyopathy, peripheral myopathy, decreased muscle tone and conduction disturbances, changes in skin appendages (hair thinning, opacification of the nails) and anemia. Cardiomyopathy in children was reported in China. It is known as Keshan disease and was attributed to a deficiency of selenium [84]. In Germany, Oster [85] observed no clinical symptoms related to selenium deficiency in the West of country. Nevertheless, they suggest that there may be a group of Germans at risk due to the low average dietary intake. This group is likely to include pregnant women, breastfeeding women, alcoholics, people with parenteral nutrition, vegetarians and people suffering from malnutrition or malabsorption. The authors also recorded serum selenium levels below the norm in alcoholics, patients with congestive cardiomyopathy, acute myocardial infarction, coronary heart disease, malignancies, liver cirrhosis and in dialysis patients.

## 6. Methods to Assess Selenium Content in Feed and Selenic Status

There are several methods for evaluating selenium in animals. It can be measured in plasma, serum, whole blood, milk or tissues such as kidney and liver. It can also be measured in urine, hair and nails. These methods are the direct ones. The atomic absorption spectrometry is the primary method used. Recently a method called "mass spectrometry inductively coupled" was developed. This technique has improved detection limits in the order of nanogram per gram of dry matter. Selenium may be also measured according to an indirect method, which is the measure of the glutathione peroxidase activity in erythrocytes. This measure does not represent the current selenic status of the animal, owing to the week’s half live of erythrocytes [86,87]. The selenoenzyme methionine sulfoxide reductase B1 (MsrB1) seems to be the most sensitive protein to a minor change in the amount of selenium dietary. For this, according to Papp *et al*. [48], it can be used as a very good marker of selenium status in humans. 

## 7. Conclusions

Selenium plays major roles in living organisms. It is present in various forms and amounts in the environment. Because of its antioxidant action and its contribution to the formation of selenoproteins, a low selenium status in the body induces a low free radicals resistance. An understanding of the mechanisms which influence the uptake and bioavailability of selenium, in both human and animals, along with knowledge of the potential sources, allows a better provision to cover the requirement of the organism with respect to this trace element. 

Different aspects of selenium metabolism remain unknown. There are factors which may reduce the body bioavailability. Absorption of selenium in sufficient amounts is important because it can be a cause of infertility in both humans and animals. Large deficiencies cause significant disfunctions and health disturbances. Soil deficiencies contribute to subsequent deficiencies in plants, animals and humans. Thus, it is necessary to find effective ways to improve the availability of selenium in food by acting on the soil-plant-animal axis.
